# Dog Breeds for the Future: Theoretical Assessment of Strategies for Enhancing the Behavioural Reliability of Existing Dog Breeds, or Developing New Ideal Urban Companion Dogs

**DOI:** 10.3390/ani16172757

**Published:** 2026-09-02

**Authors:** Péter Pongrácz, Petra Dobos

**Affiliations:** Department of Ethology, ELTE Eötvös Loránd University, Pázmány Péter sétány 1/c, 1117 Budapest, Hungary; dobospetra1@gmail.com

**Keywords:** dog breeds, public demand, amicability, companion animals, directional selection

## Abstract

The original function of many dog breeds no longer fits the requirements of an easy-going companion. Caretakers can also be overdemanding regarding their dogs’ ability to cope with societal expectations. We propose and evaluate four strategies for readjusting the dog–human relationship by highlighting their positive and negative outcomes. (1) ‘Converge’: dog breeds should be changed towards the amicable dog’s behavioural characteristics; thus, once the goal is reached, even novice dog owners can safely own almost any dog they fancy. (2) ‘Diverge’: the original behavioural/temperament traits of dog breeds should be fortified through stabilizing selection. This method emphasizes dog owners’ responsibility when choosing among the various dog breeds. (3) ‘Split’: a combination of the previous two, when clearly distinct and reliable companion and working types are selected within the same breed. (4) ‘Merge’: we should create new ‘urban dog breeds’. This method would provide ‘foolproof’ companion dog variants and at the same time relies on the prospective owners’ responsibility to choose their dog wisely. We provide a critical assessment of potential positive and negative features of each strategy, including ethical and feasibility concerns. Biologists should provide a scientific background to support a solution that favours the needs of both species in coexistence.

## Simple Summary

Dog owners face problems that relate to the behaviour of dogs in urban habitats. Poor decisions made by prospective dog owners when choosing dog breeds cause problems, too. The ‘ideal urban companion dog’ can be characterized as a low-drive, healthy, child-safe, non-barky and non-aggressive animal. In this paper we propose and assess several potential solutions for the biggest challenge that the traditional world of purebred dog breeding faces since its inception one and a half centuries ago. We argue that this change is inevitable due to societal demand for a ‘new type of companion dog’ around the world. We show that the development of various functional (i.e., behaviour-based) types of dogs, culminating in today’s breeds, has been a permanent evolutionary process, thus, the upcoming change, driven by new demands from companion dog owners and legislators, can be regarded as an organic continuation of the joint evolution of dogs and humans. We outline four possible strategies for readjusting the dog-human relationship. Whichever solution we opt for, the result will affect millions of dog owners and their societal network worldwide, and it will have a significant impact on the wellbeing of humans and the welfare of dogs for generations to come.

## 1. Introduction

### Nobody Now Who Is Anybody Can Afford to Be Followed About by a Mongrel Dog

This famous line, quoted from the prolific 19^th^-century writer William Gordon Stables in 1877, and cited from Ritvo [1], provides the perfect prelude to the topic of this paper. The Victorian era saw an unprecedented popularity explosion of leisure dog keeping among noblemen and middle-class urban citizens in Great Britain [2], and the fancy was quickly copied and adopted all around the industrialized world [3,4]. In our opinion, finding a solution to many problems that today’s dog owners are facing regarding the optimal and ethical adjustment of dogs according to the expectations of the surrounding society would be impossible without understanding how and why the concept of modern dog breeds was born. Dog owners and breeders can only work with those dogs that are the products of the organic development of pedigree dog breeding over the previous 150 years, which shaped not only their look but also defined the behavioural characteristics of the dog breeds.

Importantly, the Victorian fashion of companion dog ownership focused on a basically new ‘invention’: the purebred dog [5]. The standardization of dog breeds, known mainly as ‘land races’, became a new source of national pride [6,7]. By the beginning of the 20th century, urban (companion) dog keeping was already a popular hobby [8]. From the rural working dog types (livestock guarding, herding and multi-purpose farm dogs), and hunting dogs of noblemen, which were mostly unknown to the public a century before [9], a new genre of utility/service dogs emerged from the cataclysm of the First World War [10]. These ‘modern working dogs’ quickly became enormously popular, among the public and various armed forces, opening new avenues towards a more versatile utilization of dogs as working and sports companions for humans (e.g., [11,12]).

Studying dogs’ behaviour has become increasingly popular since the 1990s [13]. The main breakthrough for ethologists came from the notion that the natural environment for dogs is the anthropogenic niche [14,15], because this concept directed the researchers’ focus on those socio-cognitive and behavioural capacities of dogs that enabled them to adapt to the human social sphere as successful commensalists [16,17] and companions [18,19]. A systematic summary of the vastly complex field of scientific research related to dog–human interactions is beyond the scope of this paper. Among the main directions is research regarding the evolutionary origins of dogs based on molecular genetics [20,21], where comparative behavioural research, including the closest wild relative, the grey wolf (*Canis lupus*), may shed light on the possible origins of characteristic human-related socio-cognitive features of the dog [22,23,24]. A considerable segment of empirical research focuses on the role of artificial selection (ancestry: [25]; functional selection: [26]; working lines: [27]) and environmental effects (upbringing: [28]; training: [29]) on the behavioural variance among various cohorts of dogs. A quickly growing field of research uses dogs as a promising, biologically relevant model species for numerous human disorders at the level of genetics (e.g., [30]), physiology (e.g., [31]), and behaviour (e.g., [32]). Finally, the characteristics and newly emerging problems of dog–human relationships provide ample opportunity for applied ethological research as well (e.g., [33,34]). Problematic behaviours in dogs have a very complex and multi-faceted background, where the environment (e.g., socialisation: [35]; training: [36]; trauma: [37]) and the genetic background of the dog (e.g., [38]) can both influence the behavioural phenotype. In this paper, we are going to focus on those potential risk factors that stem from the mismatch between the originally sound breed-typical behaviour/temperament, the owners’ expectations, and environmental conditions (e.g., [39,40]).

Breed standards and breeding organizations still proudly refer to the original functions of the dog breeds (e.g., [41]), but new-age urban dog owners do not usually rely on those aptitudes and function-specific behavioural traits that the various working dog types have been selected for through countless generations. Although the strict isolationist principles of pedigree dog breeding effectively retained many of the main behavioural characteristics of functional working breed selection (e.g., the differences between independent and cooperative working dog breeds: [42,43]), the detectable division of many dog breeds into ‘show’ and ‘working’ lines (e.g., [44,45]), signifies the redirected aspects of directional selection compared to the traditional utilitarian methods of dog breeding.

It is safe to say that towards the 21st century, companion dog ownership became a lifestyle [46]. According to estimations, more than two hundred million dogs are being kept as pets around the world [47]. Furthermore, based on several questionnaire studies, modern dog owners increasingly regard their canine companions as family members, or even as ‘children’, instead of referring to them as simply domestic animals (e.g., [48,49]). The popularity of dog keeping has reached such levels in industrialized countries that some scientists have even started hypothesizing that companion dogs may act as surrogates for their owners, instead of them having their own children [50]. Recent research provided more elaborate detail on this matter, showing that companion dogs offered women more social flexibility and connectedness to the social environment than children; however, mothers reported higher levels of responsibility towards their children than their dogs [51]. Thus, although the human–dog emotional bond can be comparably complex and as deep as a human–human emotional relationship, the social status of companion dogs is also influenced by the family structure of their owners.

Urbanisation, a worldwide process where the density of human inhabitants shows an ever-accelerating ratio, brings new challenges for dog–human coexistence (e.g., [46]). As in ‘Western civilization’, most owned dogs are kept as companions (e.g., [52,53]), urban inhabitants demand the right to keep dogs, even if the traditional working roles of these animals cannot be fulfilled in the metropolises. Recently, city planning and human environmental research have started to assess the conditions, possibilities, and limitations of urban dog keeping among the many issues to be considered (e.g., [54,55]). While the natural needs of dogs for activity and environmental enrichment are easier to fulfil if they are kept at least partly outdoors (e.g., [56]), urban dog keeping poses serious challenges for dog owners. It is not surprising that urban planners, legislators and animal welfare experts pay close attention to many aspects of the ‘best practices’ of urban dog ownership, from the minimum number of daily walks to the maximum duration of being left alone [57], how to handle dog-derived waste [58], and the issue of nuisance barking [59].

As the behaviour of dogs represents perhaps the main synchronizing factor towards a mutually enjoyable coexistence between humans and dogs [19], many aspects of dog behaviour receive significant attention regarding their suitability for urban dog keeping. It is important to see that such characteristics that were typical and even preferred in various working dog breeds may be regarded today as unwanted for a city companion. Although nuanced details of what is an ‘ideal dog’ may vary among countries with different cultural, economic, and historical heritage [60], high activity levels, protection and guarding, being an alert and loud watchdog, and being inclined to chase and hunt are just a few examples of traits that are rarely welcome in an urban companion dog.

The recent surge of (urban) dog keeping has noticeably resulted in a novel set of demands from the ‘end users’ (dog owners), as well as from society regarding the behavioural characteristics of dogs [61]. While throughout the 20th century, advice regarding the suitability of one or the other dog breed for a particular owner remained within the scope of matching the size, temperament, and perhaps the utility of the breeds to the needs and lifestyle of the owner (e.g., [62,63]), recently, more specific expectations have been outlined that imply the need for a brand-new type of dog. The repeatedly surveyed opinion of Australian dog owners about the traits they prefer in ‘the ideal companion dog’ [64,65], crystallized the image of the ‘amicable’ dog type. This can be characterized as a low-drive, healthy, child-safe, non-barky, and non-aggressive animal that, regardless of the breed, should be the perfect companion for urban-style dog keeping. Although some subcultures still prefer characteristically non-amicable dogs, and use aggressive, menacing-looking dogs as status symbols [66], the worldwide dominant trend is leaning towards friendly and safe urban companions. Based on this, one can assume that the scene is finally set for a new era of directional selection for dog breeds that meet the demands of the largest population of prospective dog owners. Our goal with this paper is to critically assess the potential solutions to this paradoxical situation, where the traditional conservationist world of purebred dogs faces probably the biggest challenge since its origins one and a half centuries ago.

## 2. Methods: Literature Survey

We did not aim to write a systematic review; however, to better substantiate the background of our opinion piece, we ran a literature search between 20 and 23 May 2025. We explored Google Scholar and ScienceDirect using four sets of keywords: “dog breeds AND future”; “expectations AND dog breeds”; “change AND dog breeds”; and “improving dog breeds”. An additional literature search was run between 14 and 16 August 2026, on Google Scholar and ScienceDirect, where we used two new sets of keywords: “urbanisation AND dog keeping AND demands”; and “urban dog keeping AND problematic behavio(u)rs”. For each search, we examined the titles and abstracts of the first 100 hits. Based on our experience with previous literature surveys, including more hits per search resulted primarily in duplicates and thematically unsuitable items. We excluded non-peer-reviewed items, dissertations, and conference abstracts, and we kept monographs, book chapters, and peer-reviewed journal papers for further assessment. Our primary interest was in dog behaviour, breed history and dog–human interactions, with an additional focus on the specific issues relating to urban dog keeping. The final set of retained papers comprised N = 60 publications after excluding all duplicates and out-of-scope items. We further enriched the literature collection by examining the cited references in some of the best-fitting articles from our primary search.

## 3. Dog Types, Dog Breeds and Associated Behavioural Traits: A Brief Introduction to Ancestry-Based and Functional Selection

According to our current understanding, dog breeds are defined by and distinguished from each other based on three separate dimensions: (i) their ancestry (i.e., purebred dogs must be the descendants of a known and isolated group of founders); (ii) their function (i.e., original purpose-driven behaviour); and (iii) their typology/look [41,67]. It is important to understand that these three components became an indispensable part of purebred dog breeding only during the Victorian era. Closed pedigrees and the strict parameters of a dog’s appearance, as defined by breed standards, did not exist before the 19th century, and the formation of the various working dog types (landraces) was mainly driven by behaviour-based functional selection (e.g., [3]). Since then, the picture has completely changed. Although the alleged original functions of dog breeds are still highlighted in the nomenclatures of leading kennel club organizations [41], modern dog breeders put less emphasis on function-specific behaviours, which leads to increasingly uniform, uncharacteristic behavioural traits in various breeds bred mainly for conformation shows [68] and canine sports. In return, instead of the original strong directional selection towards desirable function-specific behaviours, an even more pertinent selection for the (often extreme) visual appearance of dog breeds started to characterize the last century of the dog fanciers’ world [69,70].

Although the traditional functional selection pressure, which resulted in the multitude of working-task-specific dog types (and eventually breeds), has been mostly relaxed in present times, it is easy to see that the main behavioural/temperamental traits of dog breeds can still show striking differences. Recently, a large-scale molecular genetics study could not detect breed-specific behavioural phenotypes [71], but the same paper highlighted several important genetically determined features (e.g., ‘biddability’) that overarch several dog breeds, placing them along a scale of intensity of this behavioural trait. These findings warrant the need to shift the focus from making breed-level behavioural comparisons (e.g., [72,73,74]), towards finding and investigating broader explanatory factors that can influence the socio-cognitive and behavioural phenotypes of larger clusters of breeds [41,75].

Ecological validity (i.e., why particular phenotypes would be advantageous to the investigated subjects in a given context) should always be kept in focus when we want to understand why specific behavioural phenotypes emerged in some groups of dogs, therefore functional breed selection (e.g., [43,76]) offers an especially suitable approach for testing biologically relevant differences among clusters of dog breeds. We should note that dogs’ behaviour, and particularly their social interactions with humans and other living beings, is of decisive importance in the assessment of existing dogs [40,77] and ‘engineering’ dogs for the future (e.g., [64,78]). Artificial selection efforts that shaped the various dog breed types could potentially influence fundamental socio-cognitive capacities among working dogs, depending on their intended usage. A comparison between ‘independent’ and ‘cooperative’ working dogs [79] is based on the distinct interaction styles of these dogs with their owners. Cooperative breeds originally performed their job under close human guidance with regular instructions (e.g., gundogs, herding dogs). On the other hand, hounds, livestock guardian dogs (LGDs), and terriers have been selected for tasks in which they must operate independently, either out of sight of their handlers or without being regularly instructed. From the aspect of looking for suitable dogs for modern ‘end users’ (i.e., urban companion dog owners), the functional clustering of dog breeds has a specific advantage. Cooperative and independent dog types include a wide variety of breeds, with highly variable morphological characteristics. As physical appearance is among the most important factors of preference when choosing a companion dog (e.g., [80]), the variable breed assortments with predictable behavioural characteristics within the cooperative and independent dog breed types have considerable value for prospective dog adopters. Trainability [81,82] and interactivity of dogs [83,84] are important assets for companion dog owners. In connection with these characteristics, it was found that functional breed selection affected those socio-cognitive capacities of dogs that could also play a role in everyday dog–human interactions. Cooperative dog breeds established eye-contact more easily with humans [85], they followed human-provided visual pointing more successfully [79] and they also learned better via the observation of a human demonstrator [42]. Meanwhile, it was found that independent breeds performed fewer gaze alternations when facing a difficult task [86], were more likely to steal forbidden treats when owners could not see them [87], and in general, they paid less attention to human verbal communication than cooperative breeds did [43].

Artificial selection for various working tasks in dogs has not ceased; it may have just taken different directions recently, for example: selecting dogs for novel functions such as guiding people with visual impairments [88]; serving as assistance dogs [89]; or detecting the smell of explosives [90,91]. However, the divergence between the various dog breed types has not been solely driven by the breeding efforts of humans. Based on molecular genetics, today’s dog breeds (as well as the free-ranging and feral dogs) [92,93] can be effectively clustered on the basis of their genetic distance from the common ancestor species [94,95]. Besides the functional selection-based clustering of dog breeds, ancestry-related associations between breed clades and specific behavioural traits represent another main, biologically relevant approach for behavioural investigations [41]. Regarding the main topic of this paper, we should see that the genetic distance from the hypothetical ancestor of all dogs aligns well with some of the most important socio-cognitive and behavioural characteristics of dog breeds from the aspect of their suitability to the role of a companion animal. For example, ancient breeds (such as the Chow Chow, Akita, various sled dog breeds, etc.) showed lower trainability [96] and higher stranger-directed fear [97] than the more derived (‘modern’) breed clades. Ancient breeds also showed a more consistent relationship between sociability and reactivity [23]. According to the authors, this result could be explained by the domestication syndrome (e.g., [98]), because these breeds remained relatively unaltered for a long time, they show a more uniform effect of domestication. In the more derived breeds, the two traits are more separated, which highlights that in the modern breeds [94] sociability and reactivity had different importance during selection for special working needs. Besides individual behavioural traits, the more complex problem-solving ability of breed groups can be compared based on their ancestry. By observing a human demonstrator, the modern breeds, especially utility dogs, showed higher performance in social learning than the more ancient breeds that probably were not subjected to strong directional selection for showing high interactivity with humans [25]. Obviously, when one intends to draw valid and highly predictive conclusions regarding ancestry-based behavioural characteristics of dog breeds, the broad representation of breeds and an effective comparison among more distantly related breed clades remains a vital necessity. For example, when in a survey scientists studied the trainability of only ‘ancient’ dog breeds, their alleged low performance is nearly impossible to interpret without the possibility of comparison with more derived dog breeds [99].

## 4. Behaviours That Fit, and Those That Don’t: Preferred and Problematic Behaviours and Their Association with Breeds/Types

If we consider that there are more than 400 different dog breeds recognized by the main cynological organizations, it is not an easy task to decide which breed to pick for one’s next canine companion. According to surveys, the predictable behaviour of each breed or breed type is among the most important features to consider when prospective owners choose a dog (e.g., [80]). Although dog breed descriptions (‘standards’) primarily emphasize the characteristics of the breed’s physical appearance, they usually also list the breed’s original (work-related) function and typical behaviour. However, companion dog owners, veterinarians, and kennel clubs might have very different priorities regarding favourable and suboptimal traits and aspects of breed behaviour [11,44].

For instance, in Australian society, the ideal dog could be described as safe with children, easily house-trained, healthy, obedient, and of medium size [64]. That study found differences between the preferences of owners and non-owners, as well as between genders: women preferred a calmer, non-aggressive dog, while men opted for a faithful and energetic companion. These preferences were found to be relatively stable over time (see the repeated study from Australia [65]). On the other hand, the strong fluctuation of popularity among the various breeds did not show a connection with either the consumers’ canine behavioural preferences [100] or breed characteristics such as health, longevity, aggressiveness, trainability, and fearfulness [101]. Trends of breed popularity might be affected by different types of information during different historical periods (e.g., dogs as movie stars [8], appearing in TV commercials [102], or being associated with celebrities [103], etc.).

Dog breeds were mainly developed to perform some type of function [104]. While this seems obvious regarding working dogs (e.g., herding dogs) and modern utility breeds (such as guide, police or scent detection dogs, etc.), even toy breeds were selected based on functional aspects: to provide small and easily portable companion breeds for everyday life [105]. From this perspective, it can be assumed that behaviours that characterize a breed or group of similar breeds, might have been advantageous for their original function. Therefore, in the past, humans most probably put strong directional selection pressure on these once appropriate traits [5]. The vigilant, independent, and aggressive nature of a livestock guardian dog was needed to protect the herd from thieves and predators [106]; the high activity level and boldness of terriers were preferred for dispatching pests that foraged around the hen house [107]; and sled dogs could be independent and tireless as long as they performed well in front of a sled [108].

However, these specific features that were preferred for different types of work do not necessarily correspond to the lifestyle requirements of an urban companion dog (e.g., hunting behaviour that poses a threat to other pets, wildlife, and even small children: [109]). As soon as dog ownership became a leisure activity without the need for work-related functional use, the frequent confrontation with problematic behaviours started, mainly because the behaviour and temperament traits associated with the original working function did not always suit a companion. This process is well documented through the centuries-long records from London (UK), showing how the once useful watchdogs became today’s nuisance barkers [54,110]. Of course, there might be such breed types that make it easier to become a companion dog with predominantly amicable behaviour (e.g., based on the assessment of the complex preferences of dog owners from Australia: [65]). However, there might also be such breed types that are much less suitable for becoming companions in a family environment. Problematic behaviours usually have a complex causative background (e.g., separation-related problems, [111]). Thus, the endeavour to disentangle these from each other usually requires a large sample of dogs. For instance, it was found that one main grouping factor behind problematic behaviour was the size of the dog [112]. The authors discovered that each problematic behaviour, except low trainability, occurred more frequently the smaller the dog breed was. However, dog size can be confounded with other factors such as function, keeping conditions, and longevity [113]. Hence, many of these findings would still require further investigation.

Among problematic behaviours, (the lack of) aggression towards humans is one of the most important characteristics considered by owners when they choose a companion dog [82]. For example, terriers, especially the Staffordshire Bull Terrier, are viewed as even more dangerous in the opinion of the public than they are rated by legislators [114]. These often anecdotal perceptions of some dog breeds by society would need to be further confirmed by experimental evidence. In a questionnaire study, Asp and colleagues found that dog breeds from the AKC’s working dog group showed more aggressive behaviour than the non-working breeds [75]. On the other hand, based on empirical studies, there can be a large individual variation in aggressiveness and reactivity within breeds, regardless of whether the breed is subject to legislation [115], which is a common reason why people might oppose the ban of certain dog breeds [116].

Behind both the preferred and undesirable traits, there can be additional confounding factors. More fearful dogs were found to also be more aggressive [75], which could explain some of the individual and breed-related variance of this trait. In another questionnaire study, 17 breeds were found to have significantly different noise sensitivity levels [117], however, confounders such as the dogs’ age, sex, and sensitivity to separation-related problems were also found to have an effect. The heritability of these traits should be carefully studied to determine which are the least aggressive or fearful individuals for further breeding. However, due to the complex genetic background of most behavioural traits, it might be a difficult and lengthy mission. Even if researchers find genetic associations in one breed (e.g., fearful behaviour in the Bichon Havanese, [118]), the generalization of these findings might fail when implemented in different dog breeds [118].

Proneness to aggression is a crucially important trait when the adoptability of a dog is being considered. A brief encounter with a misbehaving dog before making the decision to adopt can unjustly cause a disadvantage for dogs of the same breed [119]. Unsatisfied owners of adopted dogs might return the dog to the shelter (even without concrete evidence of problematic behaviours [120]). In turn, purebred dogs are more expensive, and owners would rather live with the inconvenience [121,122], with or without attempts to improve the quality of coexistence through training. For mixed breeds, preadoption counselling with dog experts might be a good solution to increase the success of adoption [123], as it was found that dog-relinquishing owners had higher expectations regarding desirable behaviour and their dog–human bond [40]. Moreover, behavioural assessment of dogs in the shelter can greatly improve the adoptability of dogs [124] and potentially lessen safety hazards for humans.

Problematic behaviours may also occur due to the lack of strict behavioural selection during conformation show and pet dog breeding. While in utility dog breeding, high emphasis has been put on enhancing the efficacy of selection [27], the same cannot be stated in the case of dog breeds that lack a working function [125]. Despite the increasing interest and shift in breeders’ perception of breeds [126], it has become increasingly unpredictable what kind of behaviour and temperament can be expected from a particular breed. This tendency is supported by the attitudes of some breeders who, especially in the case of highly fashionable dogs, maintain and breed further animals with well-documented, chronic health and behavioural problems (e.g., ‘trancing’ behaviour in Bull Terriers: [127]; respiratory problems in brachycephalic dogs: [128]). The high inbreeding level of some dog breeds, or breeds with low genetic variability, is not necessarily beneficial to behavioural stability either [129]. However, high levels of inbreeding can be found in breeds considered ‘healthy’ and ‘unhealthy’ as well [130], therefore the exact behavioural- and health-related consequences of inbreeding should be disentangled with targeted investigations. For instance, it was found that inbreeding can affect dog–human interaction-related socio-cognitive traits, such as aversion to humans in pointer breeds [131].

Finally, a considerable number of reportedly problematic dog behaviours can be the consequence of the owners’ expectations being unrealistic regarding preferred dog behaviours. Many people want a human-centric, strongly attached canine companion [64], however, when they leave the dog during the day, they also expect that this highly human-dependent dog should not show separation-related behaviours [132] and should not bark excessively when they return home. On the other hand, owners who prefer a healthy dog continue choosing breeds with well-known heritable diseases and anatomical problems [82,133]. How to correct and resolve these unrealistic expectations requires initiatives to help owners recognize the importance of proper breed choice (e.g., [134]).

## 5. Multi-Level Attempts to Influence/Improve Dog Behaviour and Owner-Dog Matching Success: Line Selection, Breed Crosses, Puppy Tests

Although the Victorian-era standardization of pedigree dog breeding shifted the focus towards the appearance of breeds [1], the behaviour of dogs has never become of secondary importance [75]. This is reflected not only in the continued utilization of working dogs in various traditional (e.g., livestock guarding [135] and herding [136]) and newly invented jobs (e.g., various assistance [89] and service dogs [137]), but more importantly, in the gradual and most widespread role of being a pleasant animal companion, which required the constant maintenance of behavioural soundness [78].

When we state that the natural habitat of dogs is the anthropogenic niche [18], this also means that dogs must adapt to the emerging challenges of the human environment [19]. Of course, especially in the case of purebred dogs, adaptation happens mainly via artificial selection (e.g., [138], but principally the result is the same compared to animal populations under natural selection: traits of morphology and behaviour of the breeds are being shaped according to a more optimal fit to the human-imposed requirements [41]. This remains true, no matter whether we consider the need for suitable working dogs for specific purposes [27] or for the role of being a compatible pet. Companion dog owners have explicit needs according to their lifestyle [139], fashion [102] and environmental constraints [80], but dogs’ behaviour will always remain perhaps the most important phenotype that ultimately decides whether the match between the dog and the human is going to result in success or failure (e.g., [40,123]). It is no surprise that there are plenty of methods that people have invented and continue to use with the goal of improving or securing desired behavioural traits in dogs that would make them fit for specific purposes. These procedures range from directional selection for the desired (or against unwanted) behavioural traits through planned hybridization of distinct dog breeds to interventions that target the individual dog’s behaviour via early evaluation and shorter/longer training regimes.

### 5.1. Should I Stay or Should I… Disappear—The Potential for Directional Selection of Favourable Dog Behaviours

In breed standards, dogs are typically characterized by those behavioural traits that are considered ‘type-appropriate’ and preferred according to their original purpose. Curiously enough, of all the dog behaviours that attracted scientific interest, the unwanted, or even ‘abnormal’ phenotypes were among the first that were considered to have an inherited background. Among these, we can mention the ‘unstable temperament’ of Pointers [140], or the unprovoked aggression (‘spaniel rage’) in red/golden English Cocker Spaniels (e.g., [141]). Since then, and with only a few exceptions [64,78], the context of inherited behaviours in companion dogs has still been dominated by research efforts on various undesired phenotypes [142], while applied ethologists are more likely to focus on the preferred behavioural traits of utility/working dogs [143].

In a study, based on a database of results from many thousands of dogs from the Swedish BPH test (Behaviour and Personality Description in Dogs), Tygesen et al. [144] found five behavioural factors with a weak-to-moderate genetic background: (1) playful behaviour; (2) flight and distancing behaviour; (3) sociality; (4) socially threatening and fearful behaviour; (5) submissive behaviour. Using data from eight copiously represented breeds (American Staffordshire Terrier, Golden Retriever, Labrador Retriever, Lagotto Romagnolo, Nova Scotia Duck Tolling Retriever, Rhodesian Ridgeback, Spanish Water Dog, and Staffordshire Bull Terrier), they found that flight and distancing behaviours had the strongest heritability and considered their results to be potentially useful for future breeding efforts towards less fearful and more trainable dogs.

Various forms of aggression are among the most intensively investigated undesirable canine behaviours, in which, besides the influence of the environment [38], we can find ample empirical evidence for genetic predisposition (e.g., [145,146,147]). Anxiety-related problematic behaviours are also widespread in the companion dog population [148], therefore, it is not surprising that researchers try to find genetic factors that may contribute to this feature as well (e.g., [149,150]).

The increasing availability and cost efficiency of large-scale molecular genetic techniques (such as observed- and latent-trait GWAS) allow scientists to analyse vast sample sizes of genetic material. Mahmoodi et al. [151] investigated samples from more than 40 thousand dogs across more than 100 breeds, looking for candidate genes for nine behavioural traits that originated from the widely used C-BARQ instrument (e.g., [104]). These were all behaviours that are considered ‘problematic’ in companion dogs: stranger-directed aggression, owner-directed aggression, dog-directed aggression, stranger-directed fear, non-social fear, dog-directed fear, touch sensitivity, separation-related behaviour and attachment attention-seeking. The results showed that of the 41 significant SNPs identified, 26 belonged to pleiotropic neurobehavioural genes, which underlines the complexity of potential behavioural selection efforts against the listed phenotypes.

Although thus far, the research on genetically influenced behavioural traits in companion dogs has mainly focused on the problematic phenotypes, the identification of these factors obviously can help breeders to select dogs that show more favourable behavioural patterns. Directional selection towards the ‘ideal’ companion dog within the dog breeds would be a commendable effort (as suggested by some experts, e.g., [78,125]), especially because according to the results from such breeds in which the ‘working’ and ‘show/pet’ lines were recently separated, the working-type dogs usually showed more favourable behavioural dimensions than the non-working-type dogs. D’Aniello et al. [29] found that working-type Labrador Retrievers were more attentive towards humans, and Fadel et al. [44] found that working-type Labradors and Border Collies retained more of their breed-typical impulsivity characteristics than the ‘pet-line’ dogs from the same two breeds. One solution would be to identify and pursue breeding goals with the most favourable ‘companion dog features’ (e.g., [64,65]). However, in theory, there is another method that offers a faster (but still genetics-based) recipe for reaching the sought-after, ideal companion dog behaviour: combining existing behavioural phenotypes through inter-breed hybridization.

### 5.2. Designer Dogs for ‘Good Behaviour’? Breed Crosses

Although pedigree dog breeding by definition relies on the ‘pure-blood’ ancestry of dogs according to the studbook registries, over the last 150 years, several new dog breeds have been created from already existing breeds or landraces. Perhaps the most famous example of a breed that was created by meticulous selection and crossbreeding of contemporary landraces is the German Shepherd Dog [152,153]. The Golden Retriever is another breed that has gained long-lasting popularity among fanciers and professionals, and its origin can be found in the purposeful crossbreeding of contemporary existing dog breeds in the late 19th century, such as Newfoundlands, Irish Setters, spaniels and Bloodhounds [154]. In most cases, breeders had well-defined functional goals with these pre-planned crosses, and their intention was to achieve a ‘better’ working dog breed that combined the beneficial features of the original (parental) breeds. For example, lurchers [155] are created from crosses of herding dogs and sighthounds (e.g., Border Collies and Greyhounds); the resulting hunting dog, in theory, can be better directed due to its herding dog ancestor than the more independent sighthounds [156,157].

As each of the existing dog breeds can be characterized by a combination of breed-typical behaviours (e.g., [41,75,96]), it would also be logical if dog breeders based their efforts to create ideal companion animals on combining the favourable features of different breeds. An indirect (and not recommended) example of the potential effect of crossbreeding comes from the comparative ethology of dog–wolf hybrids. Tagliarini and Temrin [158] compared the human-directed behaviour of dog–wolf hybrids to that of Malamutes, Siberian Huskies and German Shepherd Dogs. Each research subject was kept as a companion animal in the United States, and the hybrids had 11–43% ‘wolf-content’ according to DNA-testing. While the dog–wolf hybrids showed similar playfulness compared to sled dogs, and they were no more aggressive than the dogs in the study, they were significantly more fearful than any of the other tested dogs. These results show that the behaviour of hybrids in some instances can be well predicted based on our knowledge of the parental forms: wolves are undomesticated wild animals with a long history of being intensively hunted; therefore their fearfulness towards humans [159] not surprisingly prevailed in their hybrids as well. Interestingly, fearfulness/tameness was one of the clearly segregating behavioural phenotypes in the Basenji–Cocker Spaniel crosses created in the seminal project run by Scott and Fuller [160], suggesting a rather simple genetic background for this trait in dogs. However, these authors found that other capacities in their hybrid dogs, such as problem-solving ability, were more difficult to connect with the behaviour of the parental breeds.

Deliberate crosses of established dog breeds became increasingly popular for creating sought-after companion animals towards the end of the 20th century. Often dubbed as ‘designer dogs’, they are usually created for their appearance and other morphological features rather than their behaviour [70]. The most famous cluster of designer crosses is the so-called ‘doodles’, which are hybrids of Poodles resulting from crossing with other dog breeds (for example, the Labradoodle is a Poodle–Labrador Retriever cross). Poodle crosses were invented with the original intention of making a hypoallergenic coat [161], but beyond the obvious monetary interest of the breeders who create a wide array of designer dogs, allegedly positive behavioural features are also often emphasized in the case of breed crosses [70].

Owners of designer breeds most often mention the appearance of these dogs as a driving force behind their choice [161]. Besides this, the expected positive heterosis (or hybrid vigour) effect, resulting in a generally healthier dog than the traditional purebreds, is also commonly emphasized as a reason for the increasing popularity of designer dogs. To our knowledge, this assumption has not been empirically substantiated [162,163]; on the contrary, breed crosses seem to manifest the combined array of typical illnesses of their parental breeds [164]. But what about their behaviour? Apart from a very few studies (e.g., [138]), designer crosses have not yet been evaluated for their behaviour. Unlike dog breeders living a few centuries ago, or modern behavioural geneticists who purposefully target functional (working) performance [143], modern designer dog breeders apparently do not specifically aim to change the behaviour produced in these crosses relative to that seen in their originating breeds. Based on Mendelian genetics, one could assume that F1 breed crosses will exhibit ‘intermediate’ behavioural phenotypes of the parental breeds. Unfortunately, no such studies have been conducted so far on this topic comparable to the classic projects Scott and Fuller did [160], and the investigation by Shouldice et al. [165] showed no relevant difference between the C-BARQ behavioural profile [166] of Goldendoodles, Labradoodles and their parental breeds (Labrador or Golden Retrievers and Poodles). From the aspect of ‘ideal companion dog behaviour’, designer crosses that are created with thoughtful planning can have great potential value; however, a deeper understanding of the inheritance of those behaviours that play a key role (either positive or negative) in dog–human interactions would be of paramount importance.

### 5.3. Looking into the Crystal Ball of Behavioural Ontogeny: Puppy Tests

Dogs spend most of their lives as adult animals with their owners; therefore the match between the dog and the expectations/needs of humans is predominantly evaluated based on the behaviour of adult dogs. Since the seminal research of Scott and Fuller [160], the recommended time window for acquiring a puppy has been believed to be around the eighth week of life. However, because of more recent considerations suggesting that young dogs could greatly benefit from the social environment of their mother and littermates [28,167], nowadays there is a tendency to place puppies with their new owners no sooner than 10–12 weeks of age (e.g., [65]). At the same time, regardless of the future role of the dog (i.e., working dog or companion), it would be highly beneficial if: (1) based on the puppies’ behavioural traits, the ‘perfect match’ between the prospective owners’ needs and the dog could be better guaranteed; and (2) the upbringing of puppies could safeguard against problematic behaviours and generally unsound behavioural traits later in life. From this perspective, professionals and researchers have proposed and tested several methods for either improving the stimulative environment of young dogs while they are still with their mother [168] or evaluating puppies’ behaviour to forecast their suitability as adults for specific purposes/needs (e.g., [169]).

Puppy tests could help predict subsequent (adult) behavioural characteristics in at least two ways. The seemingly simpler expectation is that, based on the puppy’s temperament/behaviour, it would be possible to assign ‘the right dog to the right owner’ (e.g., [170]). These requests often go no deeper regarding dog behaviour than trying to forecast whether some puppies will become highly active/high drive, or rather laid-back specimens of their breed. According to studies, in such cases, unexperienced prospective owners would greatly benefit from simply visiting the litter of puppies with their mother present to get a glimpse of the expected behaviour of that breed and breeding line (e.g., [39,171]). However, sometimes it would be desirable to foresee much more specific information by performing behavioural tests on the puppies, such as whether they would become suitable for tasks that some of the armed forces require or to become successful candidates for sophisticated assistance-dog training [172,173,174]. Junttila and colleagues [175] found that some of their testing battery tasks were suitably predictive of adult dogs’ corresponding behavioural traits when they tested young dogs between 3 and 7 months of age. Following human pointing gestures predicted adult trainability, while performing well in a detour task predicted energy level and impulsivity, and performance in the ‘unsolvable task’ was associated with adult fear of strangers. Although in that study the young subjects had already passed the typical ‘puppy age’ when most dogs enter their new owners’ home, it seems reasonable that with properly chosen temperament and personality tests, one could find behavioural traits that stay relatively stable from juvenile age to adulthood (e.g., [176]). Unfortunately, in reality, these expectations cannot often be justified (e.g., [177,178]).

One reason for these disappointing results could be that the behavioural repertoire and typical responses of puppies can be vastly different from the adult working/sports dog typical task-related behaviours that we wish to predict (e.g., [172]). As puppies usually spend only a relatively short time at the facility where they were born, and their breeders and future owners both wish to avoid misplacement of young dogs who will later turn out to be unsuitable for the desired role, the ‘ideal’ puppy test should be feasible to perform as early as possible [170,177]. This increases the difficulty of this kind of procedure, because the younger the dog is, the simpler the behavioural reactions it can be tested for are, which do not necessarily resemble the adult behavioural pattern that needs to be predicted [172]. Thus, it is no surprise that the currently highly sought-after ‘Holy Grail’ of puppy tests (‘early—simple—highly predictive’) to the best of our knowledge, has not yet been discovered and published.

## 6. Dog Breeds in the Future: A Proposal and Evaluation of Four Potential Avenues for Their Development

In the previous chapters, we outlined that the age of modern (post-Victorian) dog breeding created not only a bewildering array of dog breeds, but towards the end of the 20th century it also helped to develop a never-before-seen popularity of companion dog keeping all around the world [102,179]. The rise of urban dog keeping has resulted in a paradoxical situation: people can now choose from hundreds of established dog breeds, which have a wide enough morphological and behavioural variability to seemingly please everyone—still, modern companion dog keeping is plagued by a fast-growing occurrence of behaviourally problematic dogs (e.g., [180,181]). The mismatch between owners’ expectations, capabilities, and the acquired dog’s temperament/behaviour likely leads to dissatisfaction and negative experiences for the owner, relinquishment of the animal, and consequently compromised human and animal welfare [181]. We have seen that in theory, there are several solutions at hand, and these could help prospective dog owners to make the best choice and know what to expect from their new canine companion. Scientific research has highlighted several behavioural and cognitive characteristics of dog breeds based on their ancestry and functional selection (for a review, see [41]), which can provide basic guidelines during the planning stage, regarding the behaviour of these breeds. Breed crosses could have some potential for pre-planning based on the behaviour of the parental breeds. In the case of separate working and pet/show/sport lines of the same dog breeds, some scientific evidence is also available to make an educated guess about their expected behaviour as well [44,182]. We should also mention the indispensable role and importance of the wide array of training methods, many of which have been found to be effective for improving the ease and quality of amicable dog–human interactions (e.g., [168,183,184]). However, these methods and information sources have been available for quite some time, without any remarkable advancement in the quality of dog–human relationships from the aspect of approaching an ‘ideal dog’.

The controversies and problems surrounding purebred dogs’ health and compromised welfare due to their extreme anatomical features have already resulted in some pioneering publications with potential solutions for future breeding efforts (e.g., [70,125]). In theory, the fundamental attributes of a highly successful urban companion dog have also been collated by several recent surveys [64,65,185]. Accordingly, dog owners would prefer an ‘amicable’ dog that is safe with children, does not show aggression towards humans and other animals, can be left at home without problems for longer periods of time, does not bark excessively, and has a laid-back temperament. Although some dog breeds’ behavioural characteristics may fall closer to this idealistic canine companion than others, we are not aware of a scientific study or applied program (except those for working dogs, e.g., [91]) that has attempted to get closer to an ‘improved’ companion dog. The public need for a scientifically grounded, large-scale program for selecting and breeding dogs with equally sound health and behavioural traits is further highlighted by such initiatives as “The Functional Dog Collaborative” https://functionalbreeding.org/about-us/ (accessed on 22 August 2026) led by distinguished researchers and canine professionals. Urged by the high occurrence and worrisome outcome of problematic (mismatched) owner–dog relationships [181] and based on the apparent inadequacy of our current possibilities towards the improvement of this situation, we believe that our societies should move towards a new era of science-based functional dog breeding. In this chapter we outline several potential avenues for developing such dogs (‘breeds’) that equally satisfy the various end users (generally: companion dog owners) and provide good welfare and safe lives for the dogs themselves. We are also going to evaluate each of the proposed strategies with the help of the SWOT (Strengths, Weaknesses, Opportunities, Threats) principle, outlined for applied ethological studies by Van Belle et al. [186]. This analytic tool provides a straightforward framework for a multiple-aspect assessment of potentially ‘competing’ solutions (e.g., [41]), thus helping to recommend considerations for future research and decisions.

**Figure 1:**
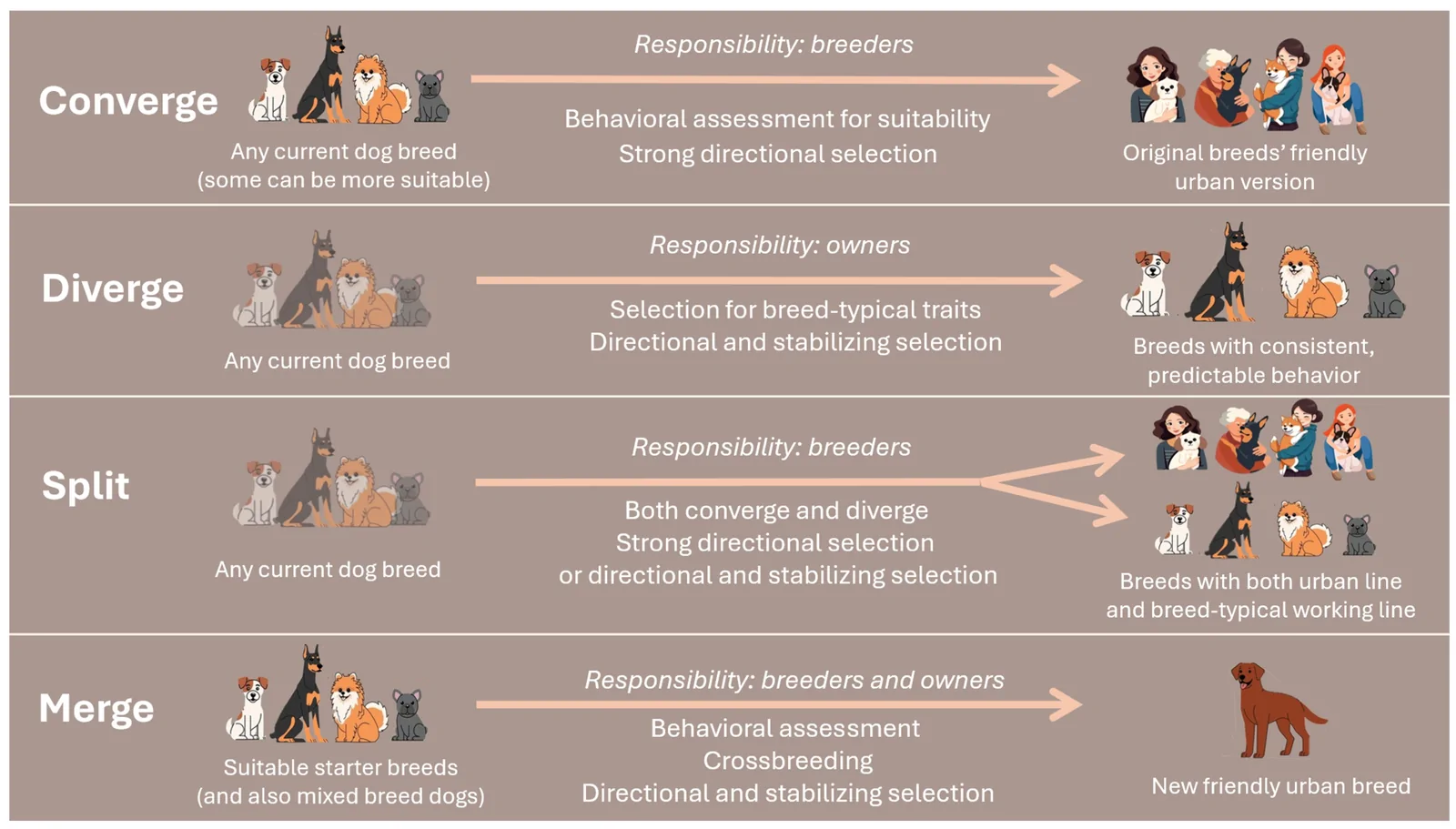
Schematic illustration of the four proposed breeding strategies to achieve predictably behaving dog breeds that are suitable for keeping as amicable urban pets. First column gives the short name of the strategy; in the next column, we indicated the initial stage of the breeding endeavour with the starter breeds. The somewhat blurred dog pictures in the case of ‘Diverge’ and ‘Split’ illustrate the less well-defined behaviour of some breeds in the beginning. The third column indicates the main steps of the breeding process. Above the arrows, we also added who has the main responsibility in securing the best choice for dog owners. The column at the right illustrates the targeted end stage of the selection process. See the detailed critical assessment of the strategies in the text.

The visual illustration of the four proposed strategies can be seen in Figure 1.

### 6.1. Strategy ‘Converge’: Converging Most Dog Breeds Towards the ‘Amicable Urban Dog’

This avenue has already been suggested by those authors who surveyed the Australian and Italian public regarding their preferences for behaviour in a canine companion [64,65,185]. Once the goal is set, in theory, breeders can exert strong directional selection on basically any breed to reach as close a match to the desired behavioural phenotypes as possible. One can assume that there will be such breeds that right from the start fall closer to some of the chosen milestones of an ideal urban dog. Others, especially such breeds that were selected/used for guarding, personal protection, fighting with other dogs/animals, or show a high drive for either cooperative (e.g., herding dogs) or individual work (e.g., terriers, Dachshunds), would initially fall short of many requirements. We should also keep in mind that beyond the results of behavioural assessments, opinions concerning a breed’s suitability for the role of being a friendly family companion can be strongly shaped by breed-related stereotypes and ad hoc encounters with positive or negative experiences [119].

Most research efforts were aimed at identifying dog breeds with the highest and lowest levels of robust phenotypes such as fearfulness, aggression, separation-related problems and excessive levels of activity (e.g., [75,187]). McGreevy et al. [112] found that in general, the smaller-bodied a dog breed was, the more frequent and intense signs of behavioural problems it showed (including fear and aggressive reactions, or problems with being separated from the owner). A Polish study highlighted that while larger-bodied dogs were more commonly reported to have problems with aggression, smaller dog breeds were reportedly more ‘hyperactive’ than others [188]. Dogs that were selected for working in close cooperation with their handler showed a stronger inclination to express signs of separation problems than the independent working breeds [132]. Ancient dog breeds (such as the Malamute, Siberian Husky and the Akita) can show strong inclinations for being aggressive with other animals, too active, and disturbingly vocal [189]. Thus, in general, each dog breed or breed type can show undesirable features from the aspect of the ideal companion dog. According to the strategy ‘Converge’ towards developing the ideal companion dog for the future, basically every dog breed requires moderate-to-strong directional selection to achieve this goal.

The main ‘philosophy’ behind this proposed avenue towards future dog breeds is that it basically puts almost all the responsibility on dog breeders and indirectly on the dogs themselves. In theory, once the required ‘user-friendly’ dogs have been created, prospective dog owners would be safe to acquire them without concern for the breed’s previously expected behaviour.

**Strength**: If a large proportion of dog breeds were to be selected to be an ‘ideal companion dog’, this would significantly elevate the general safety of ‘users’ (dog owners and their families) as well as their social environment (other people and animals). We can expect this based on the notion that human- and animal-directed aggression is one of the most serious problematic behaviours in dogs often with dire consequences (e.g., [190,191]). The widely available ‘amicable’ dogs from a large selection of breeds would positively affect the experience of dog keeping, because problematic dogs cause considerable emotional and monetary hardships in the human environment [181]. We can also expect that a more stress-free dog–human coexistence and lower occurrence of such problem behaviours such as separation-related issues would elevate the welfare of dogs as well [192]. Finally, the strength of this strategy is that we have already collected a considerable wealth of empirical knowledge about the various aspects of dog breed behaviour, which can provide a solid starting point for the outlined plans.

**Weakness**: In theory, each dog breed can have genetic flexibility that would allow directional selection towards the requirements of an amicable companion dog, and we also should not forget the positive effect of additional training by the owners as a further approximation of the desired companion dog (e.g., [33]). At the same time, large-scale behavioural comparisons among convenience sample dog breeds (e.g., [193]), as well as breed-type comparisons based either on functional breed selection [194], or breed group ancestry (e.g., [189]), showed that there are strong enough differences among the typical behaviours and socio-cognitive capacities of dog breeds that would probably make it a near-futile endeavour to select ideal companion dogs from particular breeds or breed types. Especially today, those unwanted characteristics that were once considered most useful for a given breed’s function seem difficult to ‘tone down’ by selection. For example, heightened vigilance, vocalization and territorial behaviour of guarding breeds, or the ‘hyperactivity’ of terriers, are such trademarks of the original work-directed selection that would be suboptimal in an ideal urban companion dog. Most likely, only a proportion of today’s dog breeds could provide an easy starting point for further selection towards being an ideal urban companion. As the main target traits (aggressivity/fearfulness, hyperactivity and proneness to separation-related problems) can be connected to somewhat different genetic backgrounds (e.g., [147]), the selection against each of them at the same time might be very difficult. Therefore, breeders would likely have to choose which feature of their breed would be the most important to improve first. The randomly bred mixed-breed (‘mongrel’) dogs represent another weakness in the framework of the ‘Converge’ strategy. Mixed breeds comprise a large proportion of companion dogs everywhere (for example, in the United States, nearly 50% of the dog population was reportedly non-purebred [195]). In the case of mixed breeds, we do not have extensive a priori knowledge of the dogs’ expected behavioural traits; thus using them for effective directional selection of ideal companion dogs may represent a prohibitively difficult task.

**Opportunity**: The strategy ‘Converge’ may offer a ‘last chance’ for such breeds that became unpopular or nearly extinct because of their original work function, and thus some of their behavioural characteristics turned out to be incompatible with urban companion dog keeping. Large livestock guardian breeds (LGDs) which were recently re-introduced to perform their original task of flock protection against threats posed by wolves, coyotes and golden jackals (e.g., [196]), would otherwise be difficult to integrate into companion dog status. These breeds can easily harm people due to their large size and past selection (e.g., [197,198]), and partly because of this, they are losing popularity as companions. The ‘Converge’ strategy could encourage dog breeders to develop a more compatible version of LGDs for companion purposes. The same opportunity would be true for other dog breeds with a strong propensity for being protective (e.g., Rottweiler, Boxer, German Shepherd Dog, Dobermann, etc.)—although these are not threatened by extinction, they can be problematic due to reports of their human-directed aggression (e.g., [199,200]). The opportunities of the ‘Converge’ strategy would be aided by the already ongoing processes of disruptive selection in particular dog breeds, where distinct ‘working’ and ‘show’ lines exist [44]. These lines can reveal the behavioural variability within the breeds and readily offer more raw material for further selection towards ideal canine companions. From the perspective of prospective dog owners, the ‘Converge’ strategy could also provide a favourable outcome for those that prefer such dog breeds that were originally unpopular in the community due to their negative reputation (e.g., [201]). Finally, as the strategy ‘Converge’ necessarily includes the development and application of appropriate behavioural testing, these tests could also be used for mixed-breed dogs, thus providing insight for personnel at shelters, rescue organisations, and prospective owners regarding the suitability of these dogs as urban companions.

**Threat**: The ‘Converge’ strategy indirectly poses a threat towards dog breeds that fail to show the desirable improvement towards becoming ideal companion dogs. If prospective owners generally expect predictably ‘amicable’ behaviour from the available breeds, those breeds will face a rapid loss of popularity in which the breeders are reluctant to select family-friendly lines, or the lack of the required genetic background undermines the efforts of the breeders. Such dogs would also face hardships in the case of adoption from shelters (e.g., [202]). A still-unknown magnitude of threat in the ‘Converge’ strategy would be the unpredictable loss of some breed-typical behavioural traits due to the directional selection towards companion dogs. There are indications that the ‘show lines’ of some breeds, which lacked the original work-related behavioural selection in the last decades, exhibit more similar behavioural traits to each other than their working-line counterparts [44]. Once these functional traits are lost, we may face a situation where the resulting dogs will also lose a significant part of their previous appeal. In the case of a widely successful ‘Converge’ strategy, users of original (functional) working dogs could face difficulties finding a wide enough pool of this type of their favourite breed. Another potential threat of a different nature could be the largely unexpected emergence of potentially unwanted phenotypes that have a genetic, neural or physiological connection with some of the favoured characteristics of ‘amicability’. A functional analogy is provided by research on silver foxes that have been selected for tameness only; however this directional selection also affected their morphology and non-targeted behaviours, such as their vocalizations [203,204].

### 6.2. Strategy ‘Diverge’: Lessening the Breeds’ Behavioural Unpredictability by Strengthening Their Breed-Typical Traits

Most behavioural phenotypes of the dog are strongly influenced by the environment (i.e., early socialization [28], training [205], and previous trauma [206] etc.), and epigenetics (e.g., [207]). However, recent molecular genetic advances (e.g., [208]) still suggest a detectable genetic background for signature behavioural characteristics of the main working types of dogs (such as herding, predation and trainability). The core idea behind the strategy ‘Diverge’ is not different from the generally recommended ‘best practice’ for today’s prospective dog owners: after careful examination of the possibilities and learning the existing characteristics of a dog breed, only then make an educated choice from the best-matching breeds [80]. Current research shows that dog owners indeed use a wide variety of sources of information about both the characteristics of dogs and requirements for owners (e.g., in the UK: [209]). However, as Mead and colleagues [209] noted, this information about breeds is often controversial, and according to another study, “choosing dogs on a whim” was still one of the most often reported methods used by owners [82]. If we add to this the often strongly varying behavioural profiles of dog breeds (e.g., [210]), further strengthened by the divergence due to breeding line-based disruptive selection [44], the rationale behind the ‘Diverge’ strategy becomes obvious: if dog breeds showed a more ‘clear and consistent’ behavioural profile, this predictability could positively reward those dog owners who otherwise paid keen attention to matching their needs and possibilities with the chosen breed’s characteristics. It is worth noting that the strategy ‘Diverge’ uses different breeding techniques (stabilizing selection vs. directional selection) than the ‘Converge’ strategy did and also emphasizes the main responsibilities differently. While the ‘Converge’ strategy put the main responsibility onto the breeders, the ‘Diverge’ strategy highlights the responsibility of the prospective dog owner and offers help to them by clarifying the (predictable) differences between the various dog breeds’ and breed type’ behavioural traits.

**Strength**: Perhaps the ‘Diverge’ strategy can build on the widest base of existing empirical knowledge about the various dog breeds’ behavioural characteristics. There is a rich variety of research and review papers that approached the breed-related behavioural differences from the aspect of molecular genetics (e.g., [71]), functional selection [41], or genetic distance-based ancestry [211]. Based on the practical descriptions of the original work tasks and the behavioural traits of dog breeds for fulfilling these requirements, aided by the toolkit of applied ethology in behavioural evaluation (e.g., [25,68,193]), breeders could select the most ‘breed-appropriately’ behaving subjects for further breeding. The same ‘breed-stereotypical’ knowledge could provide guidance for prospective dog owners, who would acquire such dogs from breeds that should fit their expectations.

**Weakness**: One of the main problems with the ‘Diverge’ strategy is that many first-time and experienced dog owners have inadequate knowledge about both responsible dog ownership [212,213] and the needs and characteristics of the given dog (breed). Studies show that when choosing their next dog, owners of extreme brachycephalic breeds would opt again for the same breed even though they are aware of the compromised health and welfare of these animals [133]. The attitude of such owners who are willing to accept and normalize, for example, ‘laziness’ as a breed-specific characteristic of Pugs or bulldogs, instead of regarding it as a serious consequence of cardiac and respiratory malfunctioning [214], can be a widespread phenomenon for other problematic but commonly ‘accepted’ breed-related behavioural and structural anomalies as well. Thus, the success of the ‘Diverge’ strategy mainly depends on the efficiency of owner education about what the desirable and typically expected behaviours are in each breed. A further weakness in the ‘Diverge’ strategy is the extensive variability in many breeds’ behaviour—which is further exaggerated by the rapidly diverging ‘working’ and ‘show’ lines. For example, Overall et al. [215] found that noise sensitivity showed not only breed-, but also family-line differences among Australian Shepherds, Border Collies and German Shepherd Dogs. Strong within-breed behavioural variability warrants the need for extreme caution when the ‘true’ breed characteristics are being agreed upon by breeding organizations. Finally, there is a further weakness in the strategy ‘Diverge’ as it lacks guidelines for establishing the desired, typical behavioural profile for mixed-breed dogs.

**Opportunity**: Compared to the ‘Converge’ strategy, here theoretically, each breed could have a future in its original functional form—given that society and the ‘end-users’ still need them. It is likely that most breeds can be adapted to the companion dog role if the owners receive reliable and useful guidance for their needs and characteristics, and they are also educated about the realistic assessments of their own possibilities and responsibilities as an owner [212]. The key to the success of the ‘Diverge’ strategy is making the right choices by prospective dog owners, and for these choices to impact the breeders’ efforts towards providing reliably and predictably behaving dogs according to the breed standards. If breeders who follow the recommendations of the ‘Diverge’ strategy succeed in stabilizing the breeds’ characteristic behavioural traits, it would not only be beneficial for prospective companion dog owners for making educated choices, but also the users of working dogs could benefit from it as well [27,143]. Although the genetic predisposition (heritability) of many behavioural traits is still poorly understood, and the role of ontogeny and environmental factors should not be underestimated [27], breeders and trainers of working dogs would probably have an easier task if they could start with breeds that have more clearly defined behavioural profiles. Regarding the mixed-breed dogs, as the ‘Diverge’ strategy does not specify the ubiquitous behavioural blueprint for the ideal companion dog, the wide and unpredictable variability of mongrels could also be utilized after proper behavioural profiling. Those prospective owners who prefer adoption of mixed breeds could also find better-matched companions if there was more reliable information about their main behavioural traits. Archer et al. [216] showed that the adoptability of mixed-breed dogs from shelters was more positively affected by providing temperament/behaviour profile descriptions along with pictures of the dog than providing the same photos with tentative breed labels.

**Threat**: A potential, worrisome outcome of the strategy ‘Diverge’ is the further increase in the level of inbreeding across breeds, if breeders use stabilizing selection for the behavioural traits without effective measures of preserving the genetic variability of the dogs (e.g., [217,218]). Instead of aiming towards a generic amicable behavioural profile, the strategy ‘Diverge’ emphasizes the strengthening of original, function-appropriate behaviours of the breeds. Thus, those breeds whose original employments have become obsolete, and whose typical behaviour is mainly unsuitable for companion animal purposes, would be more likely to disappear. Probably the most relevant weakness of the ‘Diverge’ strategy is the lack of a well-defined ‘ideal urban dog’, because this strategy relies on the (idealistic) image of educated and well-prepared prospective dog owners, who will choose from among the predictable and reliably behaving breeds correctly. Considering the current disappointing tendencies of highly popular dog breeds to also be aligned with a long list of morphology-, genetics-, and behaviour-based health, welfare and safety problems [101,102], it would probably be a naïve expectation to believe that dog owners would opt for such breeds that fit best their and their environments’ possibilities. Another weakness of the ‘Diverge’ strategy is that if the education of the owners and breeders is misguided or less effective, the inappropriate emphasizing of breed-specific features can have an unwanted effect of further popularizing/normalizing both the behavioural and morphological extremities of some breeds.

### 6.3. Strategy ‘Split’: The Creation of Well-Distinguishable Working- and Urban Companion Variants Within the Same Breed

If we consider the main threats of the ‘Converge’ and ‘Diverge’ strategies, it becomes clear that while the former indirectly implies the eventual loss of originally prized working dog behavioural traits, the latter likely fails to provide a wide selection of ideal city-compatible companion dog breeds. The ‘Split’ strategy can be regarded as a combination of the previous two proposed methods, with a dual aim for how to improve the behaviour of the targeted breeds. On the one hand, the goal would be to develop a reliable ‘amicable’ type; on the other hand, a reliable working line would be either restored or maintained, depending on the actual behavioural status of the existing breeding stock.

A good model for such dog breeds where the ‘Split’ strategy could provide a generally useful solution can be found in the large livestock guardian breeds (LGDs). According to recent molecular genetics results, these breeds (originally as landraces) have been developed across Europe and Asia [219] to fulfil basically the same purpose: to protect various livestock from any dangerous predators and from theft [220]. These dogs show extreme loyalty and no predatory behaviour towards livestock [135], but understandably, their aforementioned work-related behavioural traits (and large size) hardly suit urban companion status. When and where large predators (such as wolves) were hunted to extinction, LGDs lost their original jobs and their numbers, along with the knowledge of their management, were also considerably reduced (e.g., [221]). When the natural predator species started to return to their original habitats, due to the applied biological conservation programs, the need for reliable LGDs opened up again [196]. Currently, LGDs are considered not only one of the most effective but at the same time the most economical and wildlife-friendly solutions for extensive livestock management [222]. However, nowadays probably more LGDs are bred for reasons of fancy (kept for showing and/or as a companion) than for protecting livestock. Most LGD breeds are considered to be a less suitable choice for being a family companion (e.g., [223]), although their impressive appearance and size appeal to many [82]. In theory, for dog breeds such as the LGDs, where there is still a need for fulfilling the original role, which in turn is not fully compatible with the companion status, the ‘Split’ strategy could provide a satisfying solution for both types of utilization.

**Strength**: The main advantage of the ‘Split’ method is that it can be basically used on any dog breed with larger flexibility than the ‘Converge’ or ‘Diverge’ strategies. An initial population-wide assessment is needed to clarify: (1) whether there is a need for retaining the original function-specific behaviours of a breed; and (2) if this function is significantly different from the requirements of an ideal companion dog’s behavioural repertoire. The dog breeds that are undergoing the ‘Split’ method can differ in which breeding goal (‘companion’ vs. ‘original work’) requires more breeding effort to reach. Adopters of the ‘Split’ strategy can rely on the already existing breeding lines in some dog breeds (‘show’ or ‘working’ lines, e.g., in the Kelpie: [224]; in Border Collies and Labrador Retrievers: [44]).

**Weakness**: Presently it is unknown whether in some dog breeds that have already been strongly selected for a specific task/temperament, there is enough genetic variability left to reach considerable results by breeding them towards a totally different functional role. Many dog breeds suffer from a serious loss of genetic diversity (e.g., Leonberger: [225], Dobermann: [226]), which indicates that it can be problematic to find a sustainably wide genetic basis for breeding dogs for more than one behavioural type in each breed. Another general weakness of this strategy is that it does not offer a place for mixed breed dogs in the breeding or evaluation programs.

**Opportunity**: Compared to the ‘Converge’ and ‘Diverge’ methods, the ‘Split’ strategy would probably meet with the widest and easiest acceptance among the enthusiasts of the various dog breeds. Instead of moving the entire breed towards a predefined, uniform behavioural goal, with the ‘Split’ strategy it would be theoretically possible to establish the ‘companion’ and ‘working dog’ versions of the same breed. This would not only provide suitable dogs for a wider array of owners, but it would also help to maintain higher genetic diversity throughout the whole breed.

**Threat**: As the ‘Split’ strategy is intended to eliminate the problems that could arise from the adoption of either the ‘Converge’ or ‘Diverge’ methods, it has far fewer risks than those options. By allowing and encouraging the breeding of both the original breed purpose dogs and developing the new, amicable companion type, there is no threat of losing the traditional work functions or not having an urban-compatible version of the given breed.

### 6.4. Strategy ‘Merge’: The Creation of New Urban Dog Breeds

This avenue can be considered to merge the best possible outcomes of some of the previous strategies while also allowing the breeders and dog owners to opt for more traditional solutions. The main problem with the strategy ‘Converge’ was that both breeders and breed fanciers could be reluctant to accept changing their favourite breeds to a uniformly ‘amicable’ urban dog. In the case of strategy ‘Diverge’, as its success relies on the owners’ educated choices from among dog breeds that are not necessarily suitable for urban companion status, there is a good chance that they would make suboptimal decisions. The strategy ‘Merge’ emphasizes the need for newly created breeds, as the main goal of selection would be to match the requirements of an easy-going, amicable urban companion dog, most likely available in a few different sizes and appearances (e.g., coat type, ear position, etc.). By creating new breeds, fanciers of the traditional breeds would not feel that the characteristics of their favourite dogs would be threatened, and those prospective dog owners who simply need an ideal urban companion without excessive preparation and the need for training, could also find a straightforward solution.

We must admit that among the four strategies we have outlined, ‘Merge’ would probably be not only the most promising but also the most challenging. Those dog breeds that were created through planned crosses and careful subsequent selection in the first half of the 20th century were mainly built according to specific functional needs as working dogs (e.g., German Shepherd Dog, Moscow Watchdog, Czechoslovakian Wolfdog). Currently, crossbreeding between some of the popular working dog breeds sometimes occurs with the intention of maintaining robust health through higher genetic diversity (e.g., Labrador and Golden Retrievers in service and guide dog programs: [227]). So far, to the best of our knowledge, no dog breed has been selected with the goal of becoming the ideal companion dog. The recent popularity of the exponentially growing inventory of ‘designer crosses’ shows more the ingenuity of their creators as marketers to meet buyers’ preference for unusual looks than any planning for positive behavioural traits [70]. Thus, those breeders who would embark on the task of establishing new breeds based on their amicable features would need to (1) find the most promising ‘starter’ breeds (similar to the ‘Converge’ strategy); then (2) create breed crosses, and with directional selection, move towards the best-balanced features; and finally (3) stabilize the characteristics of the dogs. Only after the new ‘breed’ became reproducible with reasonably stable behaviour and appearance could its popularization among prospective owners begin.

**Strength**: With this strategy, owners could access functionally bred dogs, which would reliably show the behavioural traits that are ideal for the urban companion role. These new types would offer favourable alternatives to the traditionally preferred companion breeds, with the advantage of having multiple ancestor breeds, which may result in higher genetic diversity [228]. The high number of currently existing dog breeds could offer wide and sufficiently variable raw material for the initial selection, especially if it were to be aided by molecular genetic results identifying the most-sought (and unwanted) traits. There are already promising results regarding the heritability and genetic variability of human-directed social behaviours (e.g., [229,230]). Another advantage of the ‘Merge’ strategy is that while it could add new breeds to the market, it would leave the existing dog breeds in their current behavioural diversity (similar to the ‘Diverge’ strategy); thus, it would not raise strong resistance from breed enthusiasts who would not like to see the alteration of their breeds’ original characteristics.

**Weakness**: This strategy would probably be the most difficult to establish on a larger scale. Breeders of the new ‘amicable’ breeds could face the problem of registering their dogs, as the main kennel clubs do not embrace such endeavours. While the ‘Converge’ and ‘Diverge’ strategies did not involve crossbreeding, thus the resulting dogs could be easily registered as traditional purebreds, the success of the ‘Merge’ strategy would be highly dependent on the enthusiasm, marketing and education of both breeders and dog owners. Designer crosses are not unequivocally welcomed among dog breed fanciers and breeding organizations (e.g., [164]), and this is a warning sign for the purpose-bred new ‘amicable’ breeds, as well. A further weakness of the ‘Merge’ strategy would be its slow pace. If breeders were to aim to create ‘stabilized’ new companion breeds, it could take several generations of meticulous selection (with an average of 3 years per generation) until the behavioural traits could be stabilized. As a comparison, it took no less than 25 years to stabilize the Czechoslovakian Wolfdog after the initial and subsequent crossings with wolves [231]. Even with an optimal start and widespread education/marketing programs, we can expect that the strategy ‘Merge’ would remain a small-scale ‘connoisseur’ option for the most devoted enthusiasts for a long time. This would leave the current problems of choosing less suitable dogs as urban companions mainly intact.

**Opportunity**: By offering brand-new dog breeds that are characterized by the most suitable amicable companion dog traits, the strategy ‘Merge’ has the advantage of gaining an enthusiastic supporter base more easily than the other three strategies. The newly developed breeds would not give the impression that the selection towards urban companions would endanger the original qualities of the traditional breeds. The opportunity to educate dog fanciers about the desirable features of an urban companion dog, as well as propagating responsible dog ownership, could gain new momentum if it were connected to the introduction of the newly selected breeds. It would be easier to convince dog fanciers of the benefits of amicable companion dog behaviour when this is associated with a new type of dog, rather than argue that the long-established dog breeds should undergo fundamental behavioural changes. It would be possible to use mixed-breed dogs in the breeding program of new amicable dog types, because through behavioural testing and genetic assessments, those dogs that have some of the desired qualities could also become valuable breeding sources.

**Threat**: This strategy, even if it is successful, would provide small-scale access to the newly developed companion dogs only after a long time. The demand for the sought-after ideal amicable dogs could create an opportunity for unscrupulous breeders to offer low-quality, poorly bred dogs of questionable origin, similar to the tendencies in the breeding of today’s fashionable breeds [128]. This phenomenon would also be intensified when the positive experiences, and successful publicity about the new amicable companion breeds result in high purchase prices. We should also not forget the conservative attitude of many dog fanciers, making them reluctant to change their dog breed preferences. This is well demonstrated by the fact that despite multiple empirical results showing the complex compromised health and welfare status of extreme brachycephalic breeds (e.g., [232]), their devoted owner population resists ceasing to choose them [214]. For different reasons, supporters of adopting troubled (and mainly mixed-breed) dogs from shelters would probably also resist opting for the new companion dog breeds.

Table 1 shows the main details of the SWOT assessment for each strategy.

## 7. Discussion

Companion dogs enjoy a never-before-seen and still steadily increasing popularity around the world [53]. Besides the undeniable and mutual benefits of human–dog interactions [233], the magnitude of problems arising on both the animal and human side is also alarming [181,234]. Among the many potential influencing factors, the ever-growing proportion of urban dog keeping plays a crucial role. Many dogs seem unsuited to urban housing conditions and poorly accommodate the busy lifestyle of their owners, which comes with long periods of isolation (e.g., [64]). Parallel with this, as dog ownership became a societal norm or almost a fashion (e.g., [235]), the average knowledge base of dog owners regarding the needs of dogs and their own responsibilities remains inadequate [236]. In this opinion paper, we present a thought-provoking solution: could we amend the above-highlighted problems by placing emphasis on breeding such dogs that are either (1) more suited to the criteria of the ‘amicable urban companion’ (by following the Strategies ‘Converge’, ‘Split’ or ‘Merge’); or (2) acquire a more reliable and predictable behavioural profile according to their original breed function (Strategies ‘Diverge’ and ‘Split’).

These strategies are aimed at the modification of behavioural traits of dogs by mainly using the assets of artificial selection. Obviously, various training methods can offer faster and more readily available tools for influencing the behaviour of most dogs (e.g., [237]). We do not claim that by choosing a dog or dog breed that is the best possible match to the expectations and possibilities of the owner, this could fully substitute for the subsequent need for training and joint activities with the dog. However, there is empirical evidence that various dog breeds respond to human teaching efforts with different intensity (e.g., [43]) and efficacy (e.g., [42,194]). Dog breeds that were selected for performing cooperative tasks with humans learned more keenly and better from a human instructor [42], and they followed human communicative signals more effectively [79]; but they were also more prone to separation-related problems [132]. Furthermore, not only functional selection, but the genetic distance from the dogs’ common ancestor can also influence the dogs’ responsiveness to human presence and communication. According to a questionnaire study that used the C-BARQ instrument, primitive (ancient) dog breeds were found to be less attentive and showed weaker attachment towards their owners [238]; and based on social learning performance from a human demonstrator, modern gundogs (e.g., retrievers, pointers, spaniels) and utility breeds (German Shepherd Dog, Hovawart) were the most attentive and reliant on human behaviour [25,239]. Thus, we can conclude that currently, the various clusters of dog breeds, along with the enormous variability of mixed-breed dogs, can make it very difficult to outline a generally reliable and easy-to-use training regime for those who wish to adapt these dogs to the requirements of being an ‘ideal urban companion’. Therefore, we believe that the initiative of creating more fitting dogs (breeds) via purposeful behavioural selection would be a useful starting point for having fewer problems and more enjoyable coexistence with our companion dogs in our cities.

Clearly, none of our proposed strategies could work without the widespread, coordinated efforts of dog breeders, and preferably, international breeding organizations. Such initiatives have been suggested for supplementing high-quality detection dogs [91]; to improve canine health, well-being and welfare [240], and to solve the problems of the genetic health of purebred dogs [241]. However, regarding the organized efforts to improve the behaviour of dogs to meet public demand, we only know of a few, relatively small-scale interventions. One of these was documented in the Netherlands, where Rottweilers needed to be bred for low levels of aggression and fear to avoid legislative banning. According to the reports, when the registering of Rottweiler litters was only available if the parent dogs successfully passed the official behaviour tests, eventually the Dutch Rottweilers started to show improvements in their aggression and fear indices [200]. Breeding for a broader set of behavioural traits, such as the requirements of an ‘amicable companion dog’, would not only need clear, consensus-based target behaviours and suitable testing methods for the assessment, but also many devoted breeders aligned with a coordination council that would provide the necessary guidance and promote cooperation among the breeders. This would be especially true for the strategies ‘Converge’ and ‘Merge’. The latter is aimed at developing new amicable urban dog breeds, while the former encourages the participating breeders to perform directional selection within the existing dog breeds towards amicable traits. In these cases, success would depend on the proper and standardized behavioural evaluation of the new generations of dogs created. The strategies ‘Diverge’ and ‘Split’, would be easier, because every involved breed (or a sub-population of the breeds in the case of ‘Split’) would need to be stabilized around the typical, functional behavioural traits described in its standard and supported by practical evidence.

The Novosibirsk farm fox experiment showed that by applying strict, directional selection to a population of fur foxes (i.e., ‘wild’ animals) by always using a given generation’s tamest 10% for further breeding, a relatively fast change can be achieved towards the desirable behavioural phenotype [242]. In the case of our proposed strategies, developing amicable companion dogs at a reasonable speed could also be achieved if breeders selected only the phenotypically best specimens for further breeding. This procedure could also be aided by genetic testing if the genetic background for the desired traits were known. However, the exclusion of a larger proportion of the produced puppies from the breeding program would require administrative measures whereby those dogs with unsuitable behavioural traits could not be used later for breeding. Dog breeding is largely a leisure hobby, with a much less organized, regulated and commercialized structure than is normally the case for livestock breeding (e.g., [243]). Therefore, any of the proposed strategies for the behavioural improvement of dogs would require considerable civilian support, as well as professional guidance from the scientific community and veterinarians; predominantly the interest and enthusiasm of dog breeding organizations and the breeders themselves are imperative.

Each of the strategies would use and have consequences for mixed-breed dogs, which are not only numerous but also well-liked members of the worldwide companion dog population. The acquisition of mixed breeds can be motivated by (1) monetary reasons, (2) the wish to adopt dogs from shelters [244], and (3) by the belief that mixed breeds are healthier than purebreds [70]. There is empirical evidence that mongrels are at least as trainable and attentive as purebreds [121,245]; however, it was also shown that mixed-breed dogs show more problematic behaviours [121] and read human visual signals less effectively than purebreds do [79]. As the behavioural assessment of dogs regarding the desirable ‘amicable’ traits would be an indispensable tool in the ‘Converge’, ‘Split’ and ‘Merge’ strategies, mixed breeds could also be subjected to this procedure. The well-performing individuals could be either labelled as highly suitable candidates for the ideal urban companion role or used for the development of new dog breeds under the principles of the ‘Merge’ strategy. At the same time, as the proposed strategies are primarily aimed at the selection of improved purebred dogs (especially in the case of the ‘Converge’, ‘Diverge’ and ‘Split’ strategies), the popularizing efforts would also predominantly aim at these instead of mixed breeds. Hopefully, the pronounced need for amicable companion dogs would also have an indirect effect on stressing the importance of pre-adoption behavioural testing of mixed-breed dogs [246].

Finally, we highlight the crucial importance of the owners themselves in each of the proposed strategies for companion dog improvement. Prospective owners represent the ultimate driving force in the production and offering of dogs to the ‘market’, where breeders and shelters serve as the source. If seekers of new dogs exert their educated demand for such dogs that reliably show the traits of an amicable urban companion, the message will be clear to those on the providers’ side. Current data shows a mixed picture of dog acquisition reasons by dog owners: while the look and behaviour of the dog is important to them (e.g., [80]), just as often, they based their choice on sentimental reasons or momentary impulses (e.g., [82,133]). In the case of our proposed strategies, dog owners would need to understand that ‘amicability’ of dogs is the key component to a more enjoyable coexistence in an urban setting. With strategies ‘Converge’ and ‘Split’, we could provide a wide selection of suitable dog breeds to fulfil this need. In the framework of ‘Diverge’ and ‘Split’, dog breeds would more reliably show their original behavioural traits, thus well-educated owners could safely choose those breeds that best show the characteristics of an ideal urban companion. If the strategy ‘Merge’ were to be adopted, a whole new genre of purpose-bred companion breeds could serve the needs of knowledgeable dog owners.

Owner education programs are well known and increasingly used for various reasons. There have been initiatives for educating dog owners how to better ensure their dogs’ general health and welfare [212,247], combat canine obesity [248], or manage aggressive behaviour [249]. Lately, considerable efforts have been made to explain to fanciers of extreme brachycephalic dogs the poor welfare prospects of these breeds [250]. As the need for easy-to-live-with urban companion dogs is based on actual surveys among dog owners [64,65,185], we can be optimistic about the potential success of a campaign that would disseminate the positive features of any of the four strategies that target the development of suitable amicable companion dogs. Unlike in the case of extreme brachycephalic dogs, where the owners’ preferences for their favourite breeds have so far proven to be resistant to the grim reality of poor animal welfare (e.g., [214,250]), the need for amicable dogs in cities mirrors a public demand, which can give hope for the greater success of such initiatives.

## 8. Limitations and Potential Difficulties

In this paper we have proposed and assessed a few ideas that, in our opinion, could give coherent guidelines for organized breeding efforts, resulting in more suitable dogs for urban companion purposes. In some cases (‘Diverge’ and ‘Split’) these strategies could even provide working dogs that reliably show their original function-appropriate behavioural traits. However, the widespread practical success of these strategies heavily depends on many factors, and the proposed breeding strategies also could incur ethical concerns. We briefly mention the most relevant of these here.

### 8.1. Genetic Background of Dog Behaviour

Although there are a few promising examples of remarkably successful directional selection for sought-after behavioural traits (e.g., lessened aggression of Rottweilers in the Netherlands: [200]), to our current knowledge, most molecular genetic studies have detected a polygenic background [251,252] and widespread pleiotropy behind the behavioural phenotypes of dogs [95,151,208]. This represents serious difficulties when breeders wish to select for rather complex behavioural traits that would be preferred in an ‘ideal amicable urban dog’.

### 8.2. The Difficulty of Large-Scale Dog Breeding Programs

Problems arising from polygenic behavioural traits, low heritability and influence from the environment, can probably be overcome by using cutting-edge molecular genetics, large numbers of breeding animals, centralized breeding plans, strict selection criteria, and a standardized environment—as we can see in livestock science (e.g., [253]. With a few exceptions, dog breeding is a leisure activity, where most of the previously mentioned conditions are missing. There is hope that advances in canine molecular genetic research will provide guidance for more targeted selection. However, for successful, large-scale breeding programs a voluntary association of numerous devoted breeders would be needed, aided by such progressive breeding organizations (kennel clubs) who embrace the registration of the resulting dogs. The monetary background of such endeavours is also a crucial factor: many non-commercial dog breeders would need to finance the initial stage of the common breeding project; meanwhile the expected beneficial characteristics of the resulting dogs should be popularized among potential customers.

### 8.3. Potential Loss of Genetic Variability

Since in most dog breeds the studbooks are closed, outcrossing with breed-typical landraces, or other dog breeds is practically impossible. This, coupled with maladaptive breeding practices (e.g., using ‘popular sires’: [225]), has resulted in a significant loss of genetic diversity in many dog breeds. As some of our proposed breeding strategies necessarily include directional selection towards desired behavioural phenotypes, this may cause a further loss of genetic variability in the resulting dogs. This warrants a coordinated effort by multiple breeders who can maintain genetically separate breeding lines with the desired focus traits.

### 8.4. Ethical Considerations Regarding Shelter Dog Populations

The proposed breeding strategies in this paper focus on the improvement of dog breeds to provide predictably behaving, suitable urban companion dogs. The ‘Merge’ strategy even outlines the idea of creating new, amicable companion dog breeds through the use of behavioural assessment, targeted crossbreeding, and follow-up selection. However, we should not forget that around the world, hundreds of thousands of dogs are waiting for adoption at pounds, shelters and various rescue organizations (e.g., [254,255]. Although a considerable number of dogs are relinquished because of a behavioural mismatch with the owners’ expectations [40], and the lack of proper behaviour assessment of dogs in shelters can pose further problems [256], our moral obligation is to prioritize the adoption of those dogs placed with rescue organizations and shelters that could be safely and reliably reintegrated into the population of companion dogs. As behavioural testing for the preferred ‘amicable’ traits is an instrumental element of our proposed breeding strategies, these methods could also be implemented in the behavioural assessment of dogs placed in shelters, thus improving their chances of being adopted.

## 9. Conclusions

Our relationship with dogs is the oldest association among domesticated animals, and it has undergone constant change over the past millennia. Dogs have morphed in their form and function in parallel with the needs, preferences and possibilities of the humans living with them. However, since the advent of organized dog breeding in the 19th century, an artificially created demand for breed standardization has seemingly acted (and still tries to act) against the constant development and transformation (i.e., ‘adaptation’) of dogs according to the new requirements and needs of societies and individual dog owners. Amidst the unprecedented popularity of dog keeping in the most densely inhabited areas of our world, it is our task now to give expert guidance for the selection and nurturing of a new wave of future dog breeds. The future of dog breeds will depend on how successfully they can adapt to our urban lifestyle and the need for their original function, and it is our responsibility to shape their behavioural traits by keeping in mind the benefit of these traits for both dogs and people.

To summarize the main message and conclusion of this paper, we wish to highlight the following points:

(1) Urban dog owners would prefer ‘amicable companion dogs’, which are non-aggressive, reliable with children, have low energy levels, and can be left alone for long periods of time.

(2) Most dog breeds were originally selected to perform working tasks and therefore show several suboptimal behaviours as companion dogs in the city; meanwhile, mixed breeds have less predictable behavioural traits. However, we presume that it would be possible to genetically select for the desired ‘amicable dog’ traits in most dog breeds, aided by appropriate behavioural testing of existing and future-bred dogs as well.

(3) We propose four strategies for creating a new genre of dog breeds through purposeful breeding, with the goal of providing reliable, safe companion dogs. These dogs could hopefully satisfy owners’ needs for amicable companion animals, and they would also be better suited to life in urban settings due to their lower incidence of problematic behaviours, thereby elevating their welfare.

(4) Strategy ‘Converge’ would encourage dog breeders to exert directional selection towards the ‘amicable’ traits in most currently existing breeds. Thus, dog owners could have access to many suitable breeds with ideal behavioural traits for urban living. This strategy would be especially suitable for those breeds that already show promising companion-dog behavioural traits, and for those that show no or only a few strongly suboptimal features.

(5) Strategy ‘Diverge’ would enhance the original behavioural characteristics of the current dog breeds; thus, owners could more easily identify those breeds that are the most suitable for city living, and these dogs would reliably show the desired behaviour characteristic of their breed. This strategy would be especially advisable in the case of those dog breeds that are still commonly employed to perform their original tasks or are heavily involved in professional dog sports. Additionally, dog breeds that show mainly suboptimal behavioural traits for being an amicable urban companion would also benefit more from the ‘Diverge’ method.

(6) The strategy ‘Split’ combines ‘Converge’ and ‘Diverge’ within the breeds; thus, it helps to create a companion line and at the same time maintain a reliable working line. This strategy would be very useful for those breeds that are both wanted as companions and professional working dogs; however, the two functions require markedly different behavioural characteristics.

(7) Strategy ‘Merge’ would initiate the creation of new dog breeds that show the ideal urban companion features. For the selection, crossbreeding of current breeds, as well as mixed-breed dogs with preferred traits, could be used. This is probably the most intriguing and promising strategy, as it could perhaps be popularized most effectively. However, the creation of new companion dog breeds should be ethically justifiable due to the high number of adoptable dogs available from rescue organizations.

(8) For the success of any of these strategies, the support of many devoted breeders, breeding organizations, and expert coordinating councils would be required.

(9) As the need for easy-going, amicable urban companion dogs is based on actual demand from dog owners, we believe that the new dogs resulting from these strategies could become widely popular. However, to achieve this, whichever strategy breeders choose, widespread and well-disseminated education programs are needed for prospective dog fanciers, preferably from an early age.

## Figures and Tables

**Table 1:** The summary of the SWOT analysis in the case of each strategy proposed in this article.

	Converge	Diverge	Split	Merge
Strength	As a result, widely available amicable urban dogs from many breeds.	The widest involvement of breeds and existing breed-related knowledge.	Flexible application to most dog breeds. Already existing within-breed lines.	Offering new amicable urban dogs and leaving the original behavioural traits intact in the ancestral breeds.
Weakness	Selection for ‘amicability’ would not be feasible in many breeds. Parallel selection for several preferred traits would be difficult.	Extensive need for educating the prospective owners. Often strong within-breed behavioural variability.	In the case of already low genetic variability, the dual selection purpose might not be feasible.	Slow progress is due to the difficulty of creating new ‘breeds’ with a targeted behavioural complex. Strong resistance from traditional breeders’ organizations. Possibly small-scale output.
Opportunity	‘Last chance’ to save some rare or less popular breeds. Developing such behaviour tests that can also be used for assessing shelter dogs.	Widespread availability of reliable working dogs. With the right education, a safe choice for companion dog owners.	The highest acceptance among the various enthusiast groups. Good chance for retaining maximum genetic variability within the whole population.	The de novo created urban dog breeds would be suitable for effective education about the advantages of amicable dogs. Less friction with traditional breed enthusiasts. Easier involvement of mixed breeds for the breeding program.
Threat	Random loss of such, often-preferred behavioural phenotypes that characterized the original working dogs. Unpredictable emergence of unwanted new behavioural traits.	Disappearance of obsolete function breeds. Strong chance of loss of genetic diversity. Purpose-bred amicable companion dogs will be missing.	Not applicable, as the ‘Split’ strategy is intended to attenuate the threats that may affect the ‘Converge’ and ‘Diverge’ strategies.	High demand for the available new amicable dogs, and hence opportunistic suboptimal breeders’ practices. Ethical issues with potentially compromising the adoption of shelter dogs.
Main steps	Identify the most suitable breeds. Repeated rounds of directional selection and behavioural assessment.	Identify optimal breed functions. Repeated rounds of stabilizing selection and behavioural assessment.	Assessment of within-breed behavioural variability. Depending on the prevalence of ‘amicable’ and ‘working dog’ traits, applying disruptive selection to achieve both goals.	Behavioural and, ideally, genetic assessment of initial breeding animals to identify the most suitable dogs. Repeated rounds of directional selection and behavioural assessment.

## Data Availability

No new data were created or analyzed in this study. Data sharing is not applicable to this article.
